# Improvement of left ventricular remodeling after myocardial infarction with eight weeks L-thyroxine treatment in rats

**DOI:** 10.1186/1479-5876-11-40

**Published:** 2013-02-14

**Authors:** Yue-Feng Chen, Nathan Y Weltman, Xiang Li, Steven Youmans, David Krause, Anthony Martin Gerdes

**Affiliations:** 1 Department of Biomedical Sciences, NYIT College of Osteopathic Medicine, New York Institute of Technology, Old Westbury, NY, 11568, USA; 2 Sanford School of Medicine/University of South Dakota, Sioux Falls, SD, 57105, USA; 3 Department of Internal Medicine, Mount Vernon Hospital, Mount Vernon, NY, 10550, USA

**Keywords:** Thyroid hormone, Myocardial infarction, Myocyte, Arteriole, Collagen

## Abstract

**Background:**

Left ventricular (LV) remodeling following large transmural myocardial infarction (MI) remains a pivotal clinical issue despite the advance of medical treatment over the past few decades. Identification of new medications to improve the remodeling process and prevent progression to heart failure after MI is critical. Thyroid hormones (THs) have been shown to improve LV function and remodeling in animals post-MI and in the human setting. However, changes in underlying cellular remodeling resulting from TH treatment are not clear.

**Methods:**

MI was produced in adult female Sprague–Dawley rats by ligation of the left descending coronary artery. L-thyroxine (T4) pellet (3.3 mg, 60 days sustained release) was used to treat MI rats for 8 weeks. Isolated myocyte shape, arterioles, and collagen deposition in the non-infarcted area were measured at terminal study.

**Results:**

T4 treatment improved LV ±dp/dt, normalized TAU, and increased myocyte cross-sectional area without further increasing myocyte length in MI rats. T4 treatment increased the total LV tissue area by 34%, increased the non-infarcted tissue area by 41%, and increased the thickness of non-infarcted area by 36% in MI rats. However, myocyte volume accounted for only ~1/3 of the increase in myocyte mass in the non-infarct area, indicating the presence of more myocytes with treatment. T4 treatment tended to increase the total length of smaller arterioles (5 to 15 μm) proportional to LV weight increase and also decreased collagen deposition in the LV non-infarcted area. A tendency for increased metalloproteinase-2 (MMP-2) expression and tissue inhibitor of metalloproteinases (TIMPs) -1 to −4 expression was also observed in T4 treated MI rats.

**Conclusions:**

These results suggest that long-term T4 treatment after MI has beneficial effects on myocyte, arteriolar, and collagen matrix remodeling in the non-infarcted area. Most importantly, results suggest improved survival of myocytes in the peri-infarct area.

## Introduction

Left ventricular (LV) remodeling after myocardial infarction (MI) includes infarct expansion and hypertrophy of non-infarcted myocardium, fibrosis, LV chamber dilatation, LV functional deterioration and progression to heart failure [1-3]. The underlying cellular mechanism for maladaptive hypertrophy of the non-infarcted area includes progressive myocyte lengthening without a proportional increase in myocyte cross-sectional area [4,5]. Treatments targeting the remodeling process, such as beta-blockers and angiotensin converting enzyme inhibitors, have been shown to improve LV function as well as long-term survival. However, slow progression to chronic heart failure continues with large transmural MI as the loss of infarcted tissue and volume overload (chamber dilation) is often out of proportion to the hypertrophic response. Thus, searching for new medications that can further improve the remodeling process is critical for preventing heart failure following MI.

“Low T3 syndrome” is commonly found in patients with acute MI and other serious medical conditions. A strong association between restoration of serum T3 levels and recovery of cardiac function in patients following MI suggests that thyroid hormones play a critical role in the extent of cardiac dysfunction [6]. Animal studies have also confirmed that low thyroid status existed for up to 12 weeks following MI [7,8]. Our group has shown that treatment with triiodothyronine (T3) reduces myocyte apoptosis in the MI border area shortly after MI [9]. Based on echocardiographic measurements, other researchers also showed that longer thyroid hormone (TH) treatment can improve LV function and LV remodeling [10-12]. TH treatment promoted a beneficial change in myocyte shape in an animal model of hypertension/heart failure leading to a reduction in LV wall stress and improved function [13]. However, the effects of long-term TH treatment on myocyte remodeling following MI are unknown.

THs have been shown to induce capillary and arteriolar growth in the hypertrophic heart [14,15]. Whether they can improve arteriolar growth in the infarcted heart is unknown. Increased collagen deposition in both the infarcted and non-infarct regions is an important component of the LV remodeling process after MI. Others have reported that THs can inhibit collagen production *in vivo* and *in vitro*[16,17]. But, TH effects on collagen remodeling after MI are uncertain.

In this study, we used a MI model produced by coronary ligation in adult female Sprague–Dawley rats and treated the animals with L-thyroxine (T4) after surgery for 8 weeks. The purpose of the study was to confirm whether or not post-MI T4 treatment leads to improved LV remodeling of myocytes, arterioles, and collagen.

## Materials and methods

### Preliminary experiments

Neither the form (T3 or T4) nor an optimum administration approach for chronic treatment of animal models of cardiac disease with THs has been established. In these experiments, we employed slow-release pellets implanted subcutaneously. Pellets containing three different T3 and T4 doses each were implanted in a minimum of 5 rats per group after surgical production of MI. All rats were examined at one month post-MI. The goal was to identify a pellet dose that produced maximum improvement of LV function and improved remodeling of isolated myocyte shape with a detectible, but insignificant increase in heart rate. While the selected pellet dose would likely be too high for long-term therapy, the goal was to identify the maximum treatment benefits. The highest T3 and T4 pellet doses were well tolerated and did not lead to mortality but produced excessive tachycardia and were not considered further. Others produced no detectible effects. A 3.3 mg 60 day sustained release T4 pellet best matched selection criteria and was selected for future experiments.

### Experimental design

Adult female Sprague–Dawley rats aged 12 weeks old were used in this study. MI was produced by ligation of the left descending coronary artery [18]. Immediately following this procedure, MI survivors were randomly assigned to (1) MI group (n=24) and (2) MI+T4 group (n=23). A (3) Sham-MI group (n=21) was produced with a similar procedure except that the suture was tied loosely around the coronary artery. Thus, three groups comprised the study. T4 pellets (Innovative Research of America, Sarasota, FL; 3.3 mg, 60 days sustained release) were implanted subcutaneously into the MI+T4 group and placebo pellets were implanted into the MI and Sham-MI groups respectively immediately after surgery. Animals were housed two per cage and kept on a 12 h light/dark cycle with food and water provided ad libitum. At termination, cardiac function was assessed by echocardiography and LV catheterization for each animal in the study. All experiments and protocols were performed in accordance with the Guide for the Care and Use of Laboratory Animals (US Department of Health, Education, and Welfare, Department of Health and Human Services, NIH Publication 85–23), and approved by the University of South Dakota Animal Care and Use Committee.

### Echocardiographic measurements

A Visualsonics 660 imaging system with a 20 MHz transducer (Toronto, Canada) was used to perform echocardiography on each rat before euthanasia [19]. The rat was anesthetized under 1.5% isoflurane and two-dimensional echocardiograms were obtained from short-axis views of the left ventricle at the level of the papillary muscle tips. Two-dimensionally targeted M-mode echocardiograms were used to measure the LV dimensions in systole and diastole.

### Cardiac hemodynamic measurements

LV hemodynamics were measured with a Millar Micro-tip catheter (Millar Instruments; Houston, TX) through catheterization of the right carotid [4]. Data were recorded using an MPVS-400 Pressure-Volume System (Millar Instruments; Houston, TX). The following data were collected: heart rate (HR), LV peak systolic pressure (LVSP), LV end-diastolic pressure (LVEDP), positive/negative change in pressure over time (*dp/dt*), and TAU (time constant of isovolumic relaxation).

### Myocyte isolation

The rats in each group were separated into two groups, one for myocyte isolation and the other for whole heart tissue collection. For each MI animal, circumferential infarct length was measured just below the suture using an electronic caliper when the chest was opened.

Cardiac myocytes were isolated using a standard procedure [20]. After hemodynamic measurement, animal was deeply anesthetized, the chest was opened, and the heart was quickly removed, blotted, and weighed. The aorta was cannulated for retrograde perfusion with calcium-free Joklik media at 37°C followed by media containing 0.1% collagenase (Worthington Biomedical Corp., Lakewood, NJ). After collagenase perfusion, LV non-infarcted area + septum was separated, minced in calcium-free Joklik media, and isolated cells were poured through nylon mesh (250 μm) into 2% glutaraldehyde for cell size measurements.

### Whole heart tissue collection

After hemodynamic data were collected, the chest was opened and the heart was arrested in diastole by injection of saturated potassium chloride solution via the inferior vena cava.

The heart was then removed and cannulated through the aorta with an 18 gauge gavage needle, perfused with ice cold PBS, and subsequently trimmed, blotted, and weighed. The LV plus septum and the right ventricle (RV) were dissected and weighed. LVs were then cut into 3 pieces transversely, perpendicularly to the LV long axis. The middle slice, which was cut 1 mm below the suture, was immersion fixed in 4% paraformaldehyde for 24 h, then embedded in paraffin, and sectioned at 5 μm thickness for immunohistochemistry analysis. The basal and apical slices were frozen in liquid nitrogen and stored in −80°C until used.

### Determination of cellular dimensions of isolated myocytes

Myocytes isolated from LV non-infarcted area + septum were measured. Cell volume (V) was determined with a Coulter Channelyzer [21]. Cell length (L) of randomly selected undamaged myocytes was measured directly using microscopy and an Image-Pro Plus analysis system (Media Cybernetics, Inc., Bethesda, MD; 50 cells per sample). Cross-sectional area was calculated by dividing cell volume by cell length.

### Arteriolar staining and quantification

Paraformaldehyde fixed LV tissue sections were used. Combined Isolectin B4 (IB4, marker of endothelial cells, ECs) and α−smooth muscle actin (α−SMA, immunohistochemical marker of smooth muscle cells) labeling was used to identify arterioles. After deparaffinization, rehydration and heat-induced antigen retrieval, tissue sections were stained with FITC-conjugated IB4 (IB4-FITC; Vector Labs, Burlingame, CA; final dilution 1:200) and Cy3-conjugated α−SMA antibody (α−SMA-Cy3; Sigma, St. Louis, MO; final dilution 1:5000) in 2% BSA in TBS-T -Ca2+ for one hour, rinsed with TBS-T-Ca2+, and coverslipped with Fluoromount G (EMS, Hatfield, PA). All fluorescence images were acquired using an Olympus FluoView 1000 (FV1000) confocal laser scanning microscope (Olympus Corp., Tokyo, Japan). Myocardial arterioles were visualized by confocal microscopy at 20x magnification. On the basis of the minor diameter, arterioles between 5 and 30 μm with at least one layer of smooth muscle were classified and length density (LD) determined from each animal. Arteriolar LD (average length of arterioles/unit myocyte volume) was calculated based on the following formula: LD (mm/mm^3^) = Σ (*a/b*)/M, where *a* and *b* are the maximum and minimum external arteriolar diameters, respectively, and *M* is the solid tissue area [22]. Arteriolar LD was converted to total arteriolar length by multiplying LD (mm/mm3) by LV weight expressed in mm^3^ (~1 mg of cardiac tissue is equivalent to 1 mm^3^).

### Masson’s Trichrome staining

Paraformaldehyde fixed LV tissue sections were stained with Trichrome at the Histology Core of Sanford-Burnham Institute for Medical Research (La Jolla, CA). High resolution images were obtained through Aperio Scanscope software (Burnham Institute, La Jolla, CA) and cardiac fibrosis was quantified using Image-Pro plus (Media Cybernetics, Bethesda, MD).

### ELISA

Blood samples were drawn from opened chests via the inferior vena cava and separated into serum aliquots by centrifugation and stored at −70°C until assayed. Serum T3 and T4 levels were measured using ELISA kits according to the manufacturers’ specification. T3 and T4 kits were obtained from Monobind Inc. (human kit, Lake Forest, CA).

### Real-time quantitative PCR for gene expression

RNA from 50-100 mg of the basal part of left ventricles was extracted using the PureLink™ RNA Mini Kit (Invitrogen, Carlsbad, CA). Oligo (dT) primed cDNA synthesis was performed using RT^2^ First Strand kit (SABiosciences, Frederick, MD). Levels of αMHC, βMHC, MMP-2, TIMP-1, TIMP-2, TIMP-3 and TIMP-4 mRNAs were measured by real-time quantitative RT-PCR using the RT^2^ SYBR® Green qPCR Mastermixes with validated primers (Qiagen, Inc., Valencia, CA) and the Applied Biosystem StepOnePlus™ real-time PCR system and software (version 2.2; Life Technologies, Carlsbad, California). For RT-PCR reactions (20 μl), equal amounts (500 ng) of total RNA were used. Expression data were analyzed using SABiosciences expression analysis template in Excel (Microsoft Office 2010). The level of expression was calculated as fold change compared to sham-MI or MI control using the Ct value after normalizing with the PPIA and RPLP.

### Statistical analysis

All data are expressed as means (SD) and were compared using a two-tailed Student’s *t*-test or one-way ANOVA. In addition, a Student-Newman-Keuls or Dunnett T3 (Equal variances not assumed) post-hoc test was used to examine significant differences between groups. A value of P < 0.05 was considered statistically significant.

## Results

### Effects of T4 on heart weight and body weight

Average body weights, heart weights, and heart weight-to-body weight ratios are shown in Tables 1 and 2. Compared to the sham-MI group, both MI groups had significantly increased heart weight and heart weight-to-body weight ratios. T4 treatment significantly increased heart weight, heart weight-to-body weight ratio, and LV weight (increased by 28%, 26% and 34%, respectively) in MI animals compared to untreated MI animals. There were no significant differences in body mass among these 3 groups before surgery and at terminal experiments. MI rats with small infarcts less than 7 mm were excluded from this study. Only 2 rats in the treatment group were excluded. The average infarct length for the remaining rats was similar in both MI groups.

**Table 1:** Changes in body weight, heart weight, and infarct size

	**N**	**Body Wt1 (gm)**	**Body Wt2 (gm)**	**Heart Wt (mg)**	**Hw/BWt2 (mg/gm)**	**Infarct length (mm)**
Sham	21	239 (19)	269 (23)	824 (70)	3.1 (0.3)	
MI	24	236 (23)	273 (30)	936 (93)**	3.5 (0.4)**	10.3 (0.8)
MI+T4	23	230 (20)	272 (25)	1202 (200)** ‡	4.4 (0.7)** ‡	10.7 (1.1)
% (MI+T4 vs. MI)		−3	−0.3	28	26	4

Data presented as means (SD).

N, total number of rats (both preps); Body Wt1, body weight before surgery; Body wt2, body weight at terminal study. Heart Wt, heart weight; Hw/BWt2, heart wt-to-body wt2 ratio; *, *p* < 0.05, **, *p* < 0.01 vs. Sham rats; ‡, *p* < 0.01 vs. MI rats; ANOVA with Student-Newman-Keuls’ or Dunnett T3’s (Heart Wt and Hw/BWt2) Multiple Comparison Test.

**Table 2:** Changes in body weight, heart weight, and infarct size (whole heart tissue preps)

	**N**	**Body Wt1 (gm)**	**Body Wt2 (gm)**	**Heart Wt (mg)**	**Hw/BWt2 (mg/gm)**	**LV Wt (mg)**	**RV Wt (mg)**	**Infarct length (mm)**
Sham	9	253 (10)	268 (15)	762 (50)	2.8 (0.1)	563 (40)	138 (15)	
MI	10	249 (23)	268 (19)	906 (90)**	3.4 (0.6)*	594 (63)	205 (75)	10.5 (0.7)
MI+T4	9	243 (15)	285 (22)	1217 (135)** ‡	4.3 (0.3)** ‡	793 (185)*	304 (68)**†	11.0 (0.7)
% (MI+T4 vs. MI)		−2	6	34	26	34	48	5

Data presented as means (SD).

N, total number of rats for whole heart tissue collection; Body Wt1, body weight before surgery; Body wt2, body weight at terminal study; Heart Wt, heart weight; Hw/BWt2, heart wt-to-body wt2 ratio; LV Wt, left ventricular weight; RV Wt, right ventricular weight; *, *p* < 0.05, **, *p* < 0.01 vs. Sham rats; †, *p* <0.05, ‡, *p* < 0.01 vs. MI rats; ANOVA with Student-Newman-Keuls’ or Dunnett T3’s (Heart Wt, Hw/BWt2, LV Wt and RV Wt) Multiple Comparison Test.

### Changes of serum T3 and T4 levels after T4 treatment

Serum T3 and T4 levels are shown in Table 3. No difference was found in serum T3 and T4 levels between MI and sham-operated groups. Treatment with T4 significantly increased serum T4 levels, and tended to increase serum T3 levels but did not reach statistical significance as compared with MI and sham-MI groups.

**Table 3:** Serum T3 and T4 levels

	**N**	**Total T3 (ng/ml)**	**Total T4 (ng/ml)**
Sham	10	2.2 (0.3)	30.8 (9.8)
MI	13	2.2 (0.4)	37.3 (10.0)
MI+T4	13	2.9 (1.3)	55.3 (11.4)** ‡

Data presented as means (SD). N, total number of rats assayed.

**, *p* <0.01 vs. Sham operated rats; ‡, *p* < 0.01 vs. MI rats;

ANOVA with Student-Newman-Keuls’ Multiple Comparison Test.

### Echocardiographic changes after T4 treatment

Table 4 shows echocardiographic data. Compared with sham-MI animals, MI resulted in significantly increased LV chamber dimension during systole and diastole, decreased LV posterior wall thickness during systole, and decreased fractional shortening. No changes of the above-mentioned indices were seen with T4 treatment except for a slight but significant increase in heart rate, and a tendency for increased inter-septal thickness, which did not reach statistical significance.

**Table 4:** Echocardiographic data

	**N**	**Heart rate (beats/min)**	**IVSd (mm)**	**IVSs (mm)**	**LVIDd (mm)**	**LVIDs (mm)**	**LVPWd (mm)**	**LVPWs (mm)**	**FS (%)**
Sham	18	342 (29)	1.3 (0.3)	2.3 (0.4)	7.1 (0.4)	4.2 (0.7)	1.7 (0.4)	2.6 (0.5)	40.7 (8.9)
MI	19	342 (26)	1.2 (0.4)	1.9 (0.6)*	9.2 (0.8)**	7.2 (0.8)**	1.5 (0.3)	1.9 (0.5)**	22.4 (4.9)**
MI+T4	20	400 (68)** ‡	1.5 (0.5) †	2.3 (0.6)	9.9 (0.8)**	7.6 (1.1)**	1.7 (0.6)	2.0 (0.6)**	23.6 (6.2)**

Data presented as means (SD).

IVSd and IVSs, interventricular septal thickness in end diastole and systole, respectively; LVIDd and LVIDs, left ventricular diastolic and systolic internal diameter, respectively; LVPWd and LVPWs, left ventricular diastolic and systolic posterior wall thickness, respectively; FS, fractional shortening; **, *p* < 0.01 vs. Sham rats; †, *p <* 0.05, ‡, *p <* 0.01 vs. MI rats; ANOVA with Student-Newman-Keuls’ or Dunnett T3’s (LVIDd, LVIDs and LVPWd) Multiple Comparison Test.

### Effects of T4 on LV hemodynamics

Hemodynamic data are summarized in Table 5. MI caused a significant decrease in +*dp/dt* and –*dp/dt*, as well as an increase in LV end-diastolic pressure and TAU as compared with the sham-MI animals. T4 treatment led to an increase in ±*dp/dt* and heart rate, as well as a decrease in TAU in MI animals compared to untreated MI animals, resulting in a normalization of +*dp/dt* and TAU. No change in LVEDP was seen with T4 treatment as compared with the untreated MI group.

**Table 5:** Hemodynamic data

	**N**	**Heart rate (beats/min)**	**LVSP (mmHg)**	**LVEDP (mmHg)**	**+*dp/dt *(mmHg/sec)**	**-*dp/dt *(mmHg/sec)**	**TAU (msec)**
Sham	19	343(41)	124(14)	4.5(1.2)	8626(1830)	9958(2373)	10.2(2.3)
MI	16	335(48)	113(14)	6.1(2.0)*	6768(1465)**	5624(1232)**	13.7(2.2)**
MI+T4	17	393(74)†	127(22)	5.9(1.9)*	8862(2746)†	7058(1987)** †	10.8(2.0)‡

Data presented as means (SD).

LVSP and LVEDP, left ventricular end-systolic and end-diastolic pressure, respectively; +*dp/dt* and -*dp/dt,* maximal rate of pressure development and decline, respectively; *, *p <* 0.05, **, *p* < 0.01 vs. Sham rats; †, *p <* 0.05*,* ‡, *p <* 0.01 vs. MI rats; ANOVA with Student-Newman-Keuls’ or Dunnett T3’s (Heart rate, LVSP and +*dp/dt*) Multiple Comparison Test.

### T4 Induced beneficial changes in myocyte shape on myocytes isolated from the non-infarcted myocardium

Table 6 shows myocyte measurements. MI induced an increase in myocyte volume and length as compared with sham-MI animals. T4 treatment resulted in a further increase in myocyte volume (13% increase) due exclusively to an increase in myocyte cross-sectional area but no further increase in myocyte length as compared with untreated MI animals.

**Table 6:** LV myocyte dimensions

**Groups**	**N**	**Cell volume (μm**^**3**^**)**	**Cell length (μm)**	**CSA (μm)**
Sham	10	31516 (3484)	131.4 (3.9)	240 (26)
MI	11	36894 (3288)**	151.4 (6.2)**	244 (19)
MI+T4	10	41818 (4117)** ‡	151.9 (5.6)**	275 (28)**‡
% (MI+ T4 vs. MI)		13	0.3	13

Data presented as means (SD). N, number of rats, isolated myocyte preps;

CSA, cross-sectional area; values for cell length normalized to 1.90 μm (resting unloaded sarcomere length).

**, *p <* 0.01 vs. Sham rats; †, *p <* 0.05*,* ‡, *p <* 0.01 vs. MI rats;

ANOVA with Student-Newman-Keuls’ Multiple Comparison Test.

### Changes in left ventricular perimeters and tissue areas with T4 treatment

LV perimeter and tissue area measurements are presented in Tables 7 and 8 respectively. No significant difference was found in the inner and outer perimeters of LV, non-infarct area, and infarcted area between MI and MI+T4 groups. There was no significant difference in the percentage infarct size between these two groups either.

**Table 7:** LV areas

**Groups**	**N**	**Total tissue area (mm**^**2**^**)**	**Non-infarct area (mm**^**2**^**)**	**MI area (mm**^**2**^**)**	**Non-infarct thickness (mm)**	**Infarct thickness (mm)**	**MI area/Total tissue area**
MI	9	38.9 (4.6)	27.4 (4.5)	11.5 (2.8)	1.93 (0.36)	0.97 (0.23)	0.30 (0.07)
MI+T4	9	52.2 (12.9)**	38.6 (10.6)**	13.6 (4.2)	2.62 (0.99)*	1.26 (0.33)*	0.27 (0.08)
%		34	41	18	36	30	−10

Data presented as means (SD). N, number of rats; *, *p <* 0.05, **, *p <* 0.01 vs. MI rats; Independent Samples Student’s *t*-Test.

**Table 8:** LV perimeters

**Groups**	**N**	**LV perimeter (mm)**	**Non-infarct perimeter (mm)**	**MI perimeter (mm)**	**Infarct size (%)**
MI	9	26.4 (2.7)	14.4 (2.7)	11.9 (1.2)	46 (5)
MI+T4	9	26.4 (4.1)	15.5 (3.4)	10.9 (2.0)	42 (7)
%		0	7.6	−8	−9

Data presented as means (SD). N, number of rats. Perimeter = (outer perimeter + inner perimeter)/2.

Compared to the untreated MI group, the MI+T4 group had a significant increase in total LV tissue area (34% increase), especially the non-infarct tissue area (41% increase), as well as the thickness of both infarcted and non-infarct area (30% and 36% increase, respectively).

### The effects of T4 on arteriolar remodeling in the LV non-infarcted area after MI

**Figure 1:**
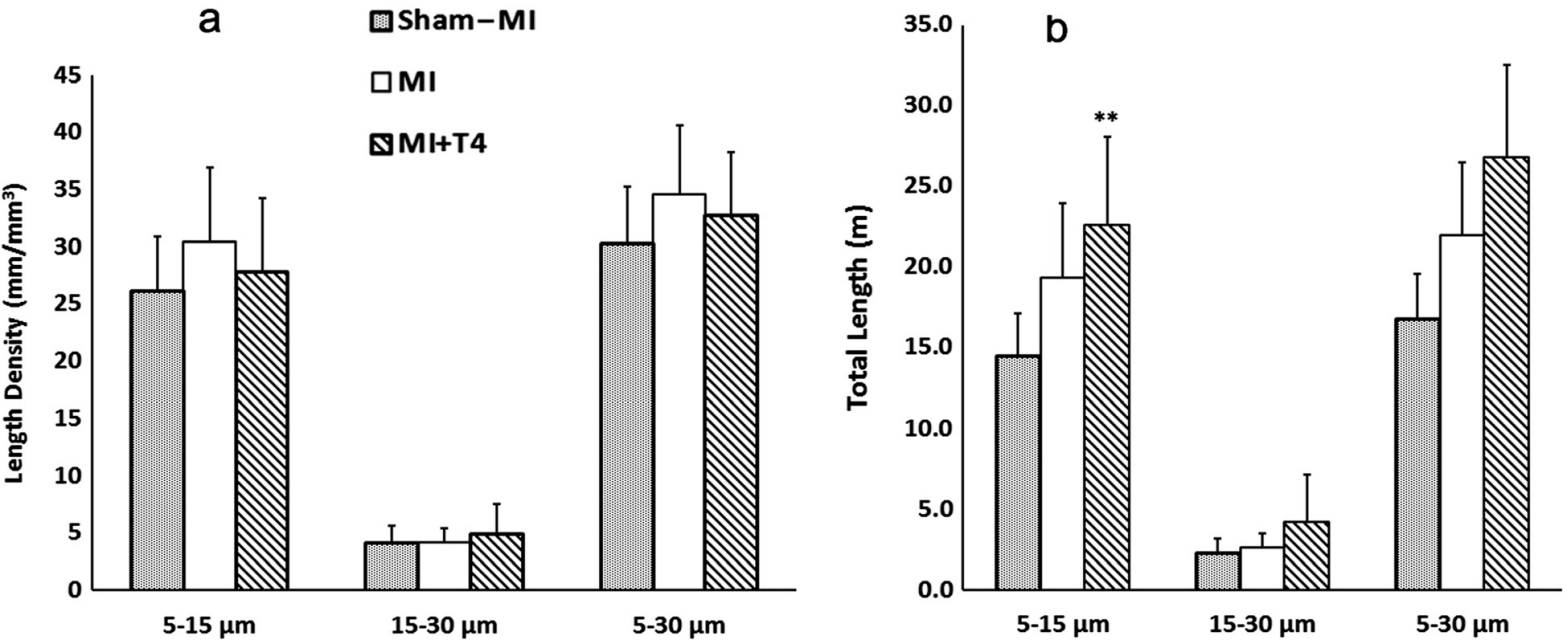
**Changes in arteriolar length density and total length in the LV non-infarcted area at 8 weeks post-MI.** (**a**) Length density; (**b**) Total length. Data are reported for arteriolar diameters in the 5–15, 15–30, and 5–30 μm ranges. Sham-MI (grey bars), MI (white bars), and MI+T4 (hatched bars). Results are mean (SD). N=7, 6 and 7 for sham-MI, MI and MI+T4 group, respectively. **, p < 0.01 vs. Sham-MI rats; ANOVA with Student-Newman-Keuls’ Multiple Comparison Test.

In smaller arterioles (5–15 μm), no change in length density was found in MI rats regardless of T4 treatment (Figure 1a), however, the total length (length density x LV weight) tended to have a stepwise increase with the LV weight increase in MI and T4 treated MI hearts (Figure 1b). No changes in length density and total length were found in larger arterioles (15–30 μm) with MI regardless of T4 treatment. Consequently, growth was in small, rather than larger arterioles.

### The effect of T4 on collagen deposition in the LV non-infarcted area after MI

**Figure 2:**
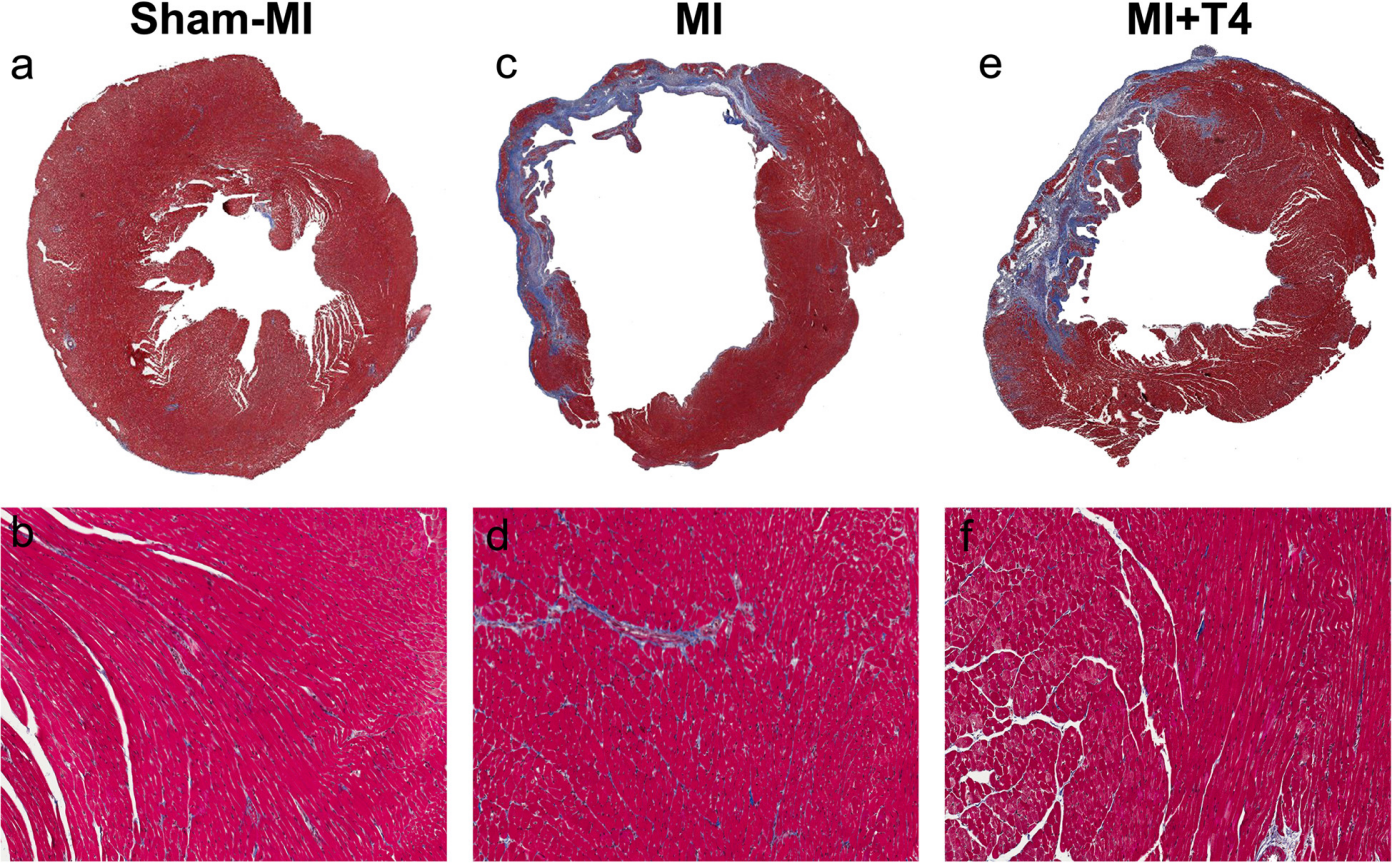
**Changes in collagen deposition in the LV non-infarcted area at 8 weeks post-MI.** Transverse myocardial sections, Masson’s Trichrome staining. (**a**, **c**, **d**) Left ventricle and septum, 0.5x magnification. (**b**, **d**, **f**) Enlarged image from septum, 10x magnification.

Collagen deposition in the LV non-infarcted area was increased with MI as compared to the sham-MI group. There was a tendency for reduction of collagen content with T4 treatment in MI animals but this did not reach statistical significance (Table 9, Figure 2).

**Table 9:** Collagen content in LV non-infarcted area

	**N**	**Collagen content (%)**
Sham	6	1.8 (0.6)
MI	5	2.9 (0.8)*
MI+T4	5	2.3 (0.9)

Data presented as means (SD). N, total number of rats.

*, *p* < 0.05 vs. Sham operated rats;

ANOVA with Student-Newman-Keuls’ Multiple Comparison Test.

### The effect of TH on the expression of MHCs, MMPs and TIMPs in the LV non-infarcted area after MI

Compared to sham-MI rats, MI caused an increase in βMHC, MMP-2 and TIMPs −1 to −4 expressions, and a decrease in αMHC gene expression. These changes were partially reversed by T4 treatment except for MMP-2, where T4 treatment tended to further increase its expression. Again, these changes did not reach statistical significance (Table 10).

**Table 10:** Fold changes in the expression of genes by RT-PCR

**Gene symbol**	**MI vs. Sham**	**MI+T4 vs. MI**
αMHC	0.60	1.17
βMHC	2.58	0.19
MMP-2	1.60	1.76
TIMP-1	2.29	0.32
TIMP-2	2.13	0.25
TIMP-3	1.91	0.67
TIMP-4	1.17	0.50

## Discussion

The T4 dose used for this study was chosen based on preliminary experiments testing several different doses of T3 and T4. A consistent beneficial effect on LV function and myocyte remodeling with a slight (borderline significant) increase in heart rate was observed with this particular dose. This dose was well tolerated by the MI rats where only 1 animal in the treatment group died after 2 days of treatment during the entire experiment (consistent with a surgical-related death). In this study, eight weeks of T4 treatment after MI significantly increased serum T4 levels and tended to increase serum T3 levels, with a reversal of the α- and β-MHC gene expression patterns caused by MI. T4 treatment increased heart weight, especially LV weight, heart weight-body weight ratio, and LV ±*dp/dt,* while normalizing TAU. Myocytes isolated from the non-infarcted myocardium showed significant lengthening with MI, and T4 treatment enhanced myocyte cross-sectional area without further increased myocyte length. There was a significant increase (34% increase) in total LV tissue area, especially in the non-infarcted tissue area (41% increase), as well as an increase in the thickness of both infarcted and non-infarcted areas (30% and 36% increase, respectively) in MI rats treated with T4. The total length of smaller arterioles ranging from 5 to 15 μm tended to increase, and collagen deposition tended to decrease with T4 treatment in the LV non-infarcted area.

Many studies have shown that either short term or long term treatment with THs starting early or late after MI can improve LV function and gross measures of LV remodeling [9,10,12,23,24]. The present study confirmed that 8 weeks of T4 treatment initiated immediately following MI can improve LV cardiac contractility and relaxation. Although no changes in LV dimension and wall thickness were appreciated with echocardiography in the present study, an increase in LV non-infarcted area thickness was found with T4 treatment in the MI rats by direct measurement of LV tissue cross-sections. In addition, myocytes isolated from LV non-infarcted myocardium were found to have an increase in cell volume after MI due exclusively to cell lengthening, which is consistent with our previous reports from rats and humans [4,5,25]. An increase in cross-sectional area without further cell lengthening was found in myocytes isolated from the same area with T4 treatment, which further increased the cell volume. This selective growth in myocyte transverse area from TH treatment is similar to results from our previous study in Spontaneous Hypertensive Heart Failure rats [13] but has never been reported in the MI model. Increased myocyte cross-sectional area contributed to increased wall thickness in the LV non-infarcted area with T4 treatment, a change that should reduce wall stress and improve LV function. It should be noted that T4 treatment led to only a 13% increase in cell volume (exclusively from increased cross-sectional area), which cannot account for the 34% increase in LV weight and the 41% increase in the non-infarct tissue area. Since the Coulter Channelyzer method offers precise measurements of isolated myocyte volume when high quality cells are assessed and whole tissue changes can also be precisely collected, these combined data indicate that more myocytes were present in the non-infarct area with T4 treatment as compared to untreated MI rats. This finding, that isolated myocyte volume accounted for only ~1/3 of the increase in muscle mass from TH treatment, was consistently observed in other preliminary experiments. We previously reported that TH treatment immediately following MI reduced the expression of markers of apoptosis in myocytes in the border area [9]. New data provided here suggest that the previously observed TH-mediated inhibition of apoptosis led to increased preservation of myocyte number in the border zone of the non-infarcted tissue area.

Small arterioles are very important for myocardium perfusion and oxygen supply. The pro-angiogenic effect of THs has been demonstrated in different models in which the growth of capillaries and arterioles were observed [14,15,26]. A stepwise increase in the total length of smaller arterioles (5 to 15 μm) was found following MI and T4 treatment, indicating a parallel growth of small arterioles along with myocyte hypertrophy, which would help maintain blood supply to the hypertrophic heart.

Increased collagen deposition observed in the non-infarcted area here was also found by others [27,28]. Eight weeks T4 treatment following MI tended to decrease collagen deposition in the non-infarct area, which might contribute to the improvement of LV relaxation. The anti-fibrotic effect of TH has been documented in culture conditions as well as in TH-induced hypertrophic hearts which was mediated by either decreased collagen production or increased collagen degradation [16,29,30]. The collagen content in the infarcted area was not measured in this study. However, we observed no increase in cardiac deaths in the T4 treated group during the early stage of post-MI remodeling, indicating that T4 treatment did not significantly interfere with the scar formation process.

Matrix metalloproteinases (MMPs) and tissue inhibitors of metalloproteinases (TIMPs) are critical in extracellular matrix remodeling by degrading certain components, regulating cell proliferation, migration, differentiation, and apoptosis as well as angiogenesis. More specifically, studies have shown that MMP-2 promotes angiogenesis by releasing angiogenic factors from the matrix, while TIMPs −1 to −3 can inhibit vascular endothelial cell migration and/or angiogenesis through releasing anti-angiogenic factors from matrix or interacting with angiogenic growth factor receptors [31,32]. With regard to cardiomyocyte hypertrophic remodeling, TIMPs −1 and −3 have been shown to exhibit an inhibitory effect [33-35]. Little is known about the role of TIMP-4 on cardiac remodeling besides its inhibitory effects on MMPs. The expression of MMPs and TIMPs following MI has a temporal and spatial pattern, where in the post-MI late stage, MMP-2 expression in the remote and border zone varied in different studies, TIMPs −1 to −3 expression was reduced in both remote and border zone, and TIMP-4 was unchanged in the remote myocardium but decreased in the border area [36-39]. In the present study, there was a tendency for increased MMP-2 and TIMPs −1 to −3 expression in the non-infarcted myocardium (includes both remote and border zone) late after MI. With T4 treatment, there was a tendency for further increase in MMP-2 expression but a decrease in TIMPs −1 to −4 expressions in the above-mentioned area. The difference in the findings of MMP-2 and TIMPs expression in these MI studies might be due to animal model differences and different methods of tissue sampling. No report can be found regarding the effects of THs on MMPs and TIMPs expression in the non-infarcted area during late stage post-MI LV remodeling. However, Ziegelhoffer-Mihalovicova *et al.* reported that T3-induced cardiac hypertrophy was not accompanied by cardiac fibrosis but an increase in MMP-2 and TIMP-2 expression [40]. Ghose Roy *et al.* found a reduction in collagen I and III in cardiac tissue with an increase in MMP-1 activity and a decrease in TIMP-3 and TIMP-4 expression in T3-induced cardiac hypertrophy [30]. Thus, the long-term effects of T4 on myocyte remodeling and arteriolar growth in the non-infarcted area following MI might also relate to its actions on the expression of MMPs and TIMPs, which requires additional investigation to verify.

A number of animal studies in recent years have demonstrated a decline in tissue T3 levels after MI due to increased cardiac expression of the D3 deiodinase, which converts T4 to rT3 and T3 to T2 [41,42]. A report showing worse outcomes in post-MI patients with elevated serum rT3 levels suggests a similar process occurs in humans [43]. Based on this finding, one might expect T3, rather than T4, to be more effective in restoring cardiac tissue T3 levels post-MI. While the increase was not statistically significant, serum T3 levels were 32% higher in T4-MI rats compared to MI or sham groups. This trend suggests that peripheral conversion of T4 to T3 occurred as a result of elevated serum T4 and likely provided additional T3 for cardiac myocyte uptake.

Regulation of intracellular T3 is a complex process involving availability of free T3 in serum, as well as thyroid hormone membrane transporters and intracellular thyroid hormone binding proteins in the target cells. There is currently no information available regarding changes in thyroid membrane transporters and intracellular thyroid binding proteins in heart disease. Nonetheless, improvements in cardiac remodeling and function observed here suggest increased availability of T3 in the myocytes of treated rats. Now that important cardiac benefits of "supraphysiological" doses of THs post-MI have been demonstrated here and previously [12], a critical question remains unanswered. Can the functional and remodeling benefits of T3 or T4 treatment of MI be safely and effectively implemented in patients without adverse effects?

TH signaling is a complex and poorly understood process involving genomic and non-genomic signaling mechanisms [44]. Genomic signaling involves T3 binding to thyroid nuclear receptors associated with the thyroid response element of targeted genes. Involvement of many thyroid receptor isoforms, co-activators, and co-repressors contribute to the complexity of genomic signaling. Many signaling pathways have been implicated in non-genomic TH signaling, including MAP kinases, PKC, and Akt. Work from our lab has identified a particularly important role for Akt signaling in myocyte and vascular remodeling [45-47].

## Conclusions

T4 treatment of rats with MI led to significant improvement in LV function. The current study also used combined whole tissue and isolated myocyte preps to investigate myocyte remodeling from T4 treatment of MI. Myocyte shape improved but accounted for only 1/3 of the T4-induced increase in muscle mass, suggesting improved myocyte survival. T4 treatment led to increased thickness of the non-infarcted and infarcted segment length, changes that should reduce wall stress. Small arterioles increased in proportion to muscle mass, suggesting T4 induction of physiologic vascular growth. There was also a trend toward reduced LV fibrosis with treatment. This study provides further evidence suggesting potential benefits from TH treatment of MI.

## Competing interests

The authors declare that they have no competing interests.

## Authors’ contributions

YFC was the Project leader and performed surgeries, collected hemodynamic measurements, sample collection, data collection, statistical analysis, and manuscript preparation. NYW conducted arterial quantification and PCR. XL collected LV perimeter and tissue area measurements. SY conducted ELISA assays. DK collected comprehensive myocyte measurements. All work was conducted in the laboratory of AMG under his supervision. All authors read and approved the final manuscript.
